# Prevalence and characteristics of adults avoiding gluten without celiac disease: a long-term population-based follow-up study

**DOI:** 10.1186/s12876-025-03799-x

**Published:** 2025-03-25

**Authors:** Miska Tiainen, Kalle Kurppa, Tuija Jääskeläinen, Niina Kaartinen, Heini Huhtala, Katri Kaukinen, Juha Taavela

**Affiliations:** 1 Celiac Disease Research Center, Faculty of Medicine and Health Technology, Tampere University and Tampere University Hospital, Elämänaukio 2, Tampere, Finland; 2 Tampere Center for Child, Adolescent, and Maternal Health Research, Department of Pediatrics, Tampere University, Tampere University Hospital, Tampere, Finland; 3 University Consortium of Seinäjoki, Seinäjoki, Finland; 4 Finnish Institute for Health and Welfare, Helsinki, Finland; 5 Faculty of Social Sciences, Tampere University, Tampere, Finland; 6 Department of Internal Medicine, Tampere University Hospital, Tampere, Finland; 7 Department of Gastroenterology, Tampere University Hospital, Tampere, Finland

**Keywords:** Non-celiac gluten sensitivity, Gluten avoidance, Celiac disease, Gluten, Transglutaminase

## Abstract

**Objective:**

Nationwide prevalence studies on people avoiding gluten without celiac disease (PWAG) are lacking, and in particular, long-term follow-up studies are unavailable. We aimed to evaluate the prevalence, incidence, and characteristics of PWAG in a population-based cohort in 2000 and 2011.

**Methods:**

Health and diet-related data were collected in nationwide Health 2000 and 2011 surveys, which comprised 5,777 and 3,866 individuals, respectively, representing 2,682,733 and 1,967,876 Finnish adults. Serum samples were taken for the measurement of transglutaminase autoantibodies. In total 3,296 individuals participated in both surveys, forming a prospective cohort. PWAG refers to subjects avoiding gluten without celiac disease or positive autoantibodies. Psychological health was assessed with General Health Questionnaire and the Beck Depression Inventory.

**Results:**

The prevalence of PWAG increased significantly from 0.2% (2000) to 0.7% (2011) (*p* < 0.001), with the highest prevalence (1.3%) detected in individuals > 70 years old. An annual incidence rate of 42 (95% confidence interval 25–71) per 100,000 persons was noted. The PWAG group was more likely to maintain additional special diets than those not avoiding gluten, including e.g. lactose-free diet (41.7% vs. 12.0% in 2011, *p* < 0.001) and food restriction for allergy (12.5% vs. 3.0%, *p* = 0.007). Beck Depression Inventory indicated more depression (*p* = 0.023) among PWAG in 2000, while no difference was seen in 2011 or in General Health Questionnaire. Celiac disease-related risk factors, including female gender, anemia, autoimmune diseases or antibody levels near the upper limit of normal in 2000, did not predict later gluten avoidance.

**Conclusions:**

The prevalence of PWAG multiplied over a decade, reaching 0.7% in 2011 in Finland. The PWAG group maintained more likely additional dietary restrictions than those not avoiding gluten and had signs of psychosocial burden. No predicting factors for the condition were identified.

## Introduction

In non-celiac gluten sensitivity, intake of dietary gluten may cause symptoms in individuals without evidence of celiac disease or wheat allergy [1]. The exact cause of non-celiac gluten sensitivity is unclear, but one common hypothesis is that gluten could increase intestinal permeability, leading to detrimental interactions with the gut microbiome and mucosal cells under epithelial lineage [1, 2]. However, the role of gluten as a causal agent has also been questioned, as other wheat ingredients could also be detrimental to these individuals [2]. Besides to symptom relief, individuals without celiac disease may adopt a gluten-free diet for other reasons, such as perceived health benefits [2, 3]. Hence, as subjects may avoid gluten due to various reasons this whole group is commonly referred as “people without celiac disease avoiding gluten” (PWAG) [4]. The prevalence of PWAG has been analysed in a limited number of studies yielding variable results, with estimates reaching up to 15%, although this may partially reflect sampling bias [5–7]. While there are some data suggesting an increasing prevalence of PWAG [8], longitudinal evidence from unbiased, population-representative cohorts is lacking [4]. 

Characteristics PWAG and potential predictive factors for gluten avoidance are also poorly known and the few published studies have shown inconsistent results [6, 8]. Some reports suggest an overrepresentation of autoimmune diseases in these individuals, but these studies have generally been small [9, 10]. Additionally, the possibility of undiagnosed celiac disease may have been overlooked, and diagnosing subjects already on a gluten-free diet can often be complicated [11]. Improved characterisation of PWAG and its possible associated risk factors could provide valuable insights into the pathogenesis, natural history and potential complications of the condition, as well as enhance case-finding and long-term follow-up by healthcare providers.

We aimed to study the prevalence and characteristics of PWAG in two large, nationally representative population samples of adults collected in 2000 and 2011. Comprehensive demographic and clinical data were obtained with celiac disease serology allowing the exclusion of diagnosed celiac disease patients and those being positive to celiac disease antibodies in this cohort. A prospective sub-cohort of individuals that participated in both 2000 and 2011 allowed us to analyse incidence and possible risk factors for developing the condition.

## Materials and methods

### Patients and study design

The study was conducted in collaboration between Tampere University and the Finnish Institute for Health and Welfare (THL). It was based on two nationally representative health surveys carried out by THL in 2000 and 2011 [12, 13]. The main aim of these extensive surveys was to provide up-to-date data on major public health problems in Finland. The surveys comprised a probability-clustered sampling and weighting scheme that estimates health statistics representative of the same-aged Finnish population at that time [13–15]. 

In Health 2000, a total of 8028 adults aged 30 years or more living in mainland Finland were invited to participate in a health examination, interview, and blood sampling and complete quality of health and nutritional questionnaires. Only individuals with data available on possible previous celiac disease and/or gluten-free diet who had provided sera for the celiac autoantibody analyses were included in this study. To facilitate the two-stage stratified sampling, the sample frame was stratified regionally according to five university hospital regions (strata): namely those of Helsinki, Turku, Tampere, Kuopio and Oulu. In the first stage of sampling, 80 health centre districts were sampled as a cluster. The sample size for each health centre district was proportional to its proportion of the population. In the second stage, systematic random sampling was used to draw the sample from each health centre district using the nationwide population register. All living members of the original Health 2000 sample were reinvited to participate in Health 2011 and this group comprised the prospectively followed-up sub-cohort for the present study. The unweighted participation rate at any stage of the survey was 93% in Health 2000 and 73% in Health 2011 [12, 13]. Additional samples of younger adults were also invited in both studies, but they only completed a postal survey and were thus not included in the present analysis.

**Fig. 1:**
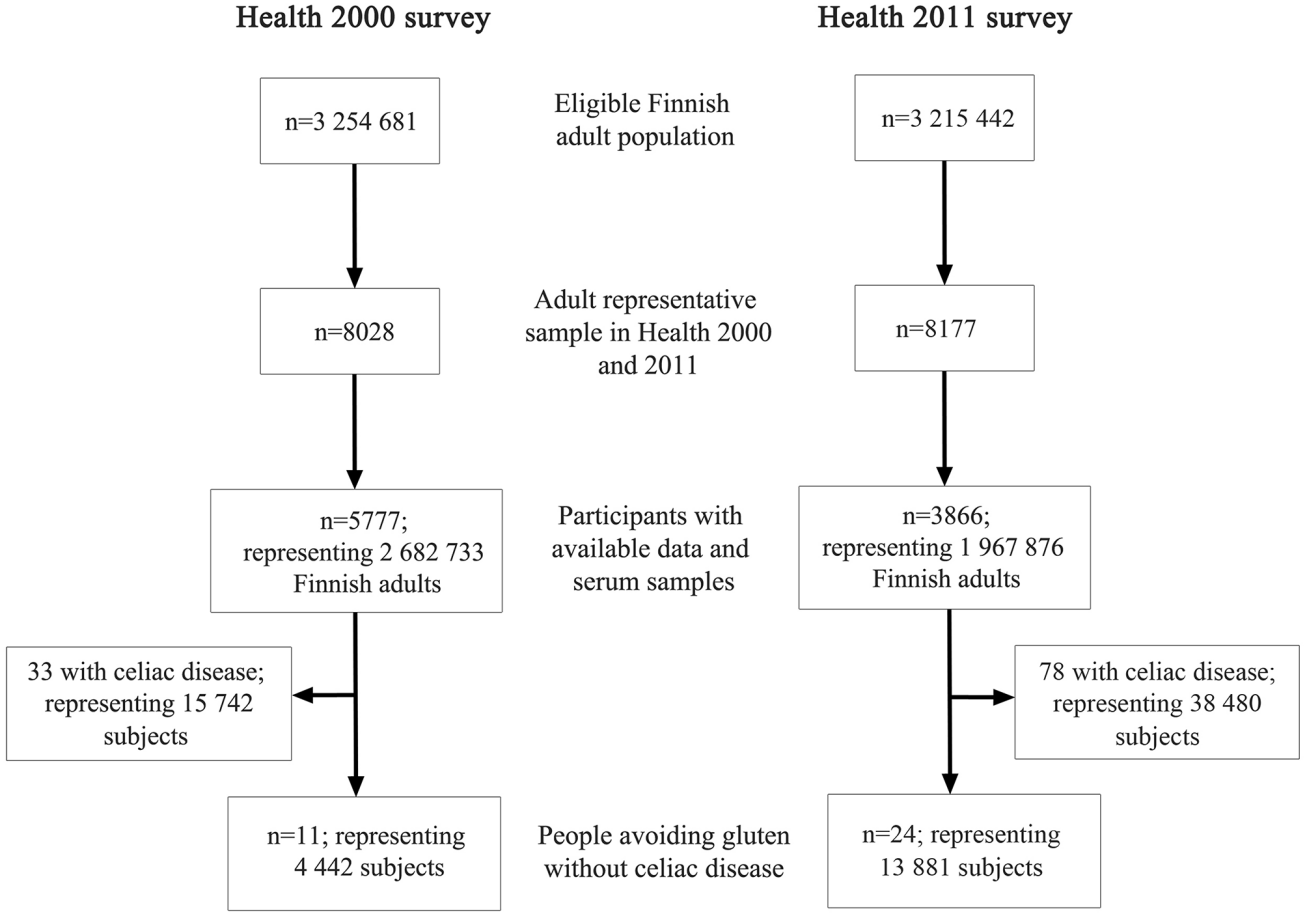
Flowchart of the study. The study cohorts, that represented the population of Finland at respective time points, consisted of individuals who participated in Health 2000 and 2011 surveys and had available data on celiac disease and gluten-free diet and had provided serum sample for the measurement celiac disease antibodies. The diagnosis of celiac disease was based on patient´s responses and supplementary medical registry data with addition of those subjects having positivity in both tissue transglutaminase and endomysial antibodies. “People avoiding gluten without celiac disease” refers to subjects who avoid dietary gluten without celiac disease diagnosis and had negative celiac disease antibodies

The eligible population in Health 2000 was 3,254,681 and in Health 2000 3,230,382 individuals, and the corresponding representative samples 8,028 and 8,177 individuals, respectively (Fig. 1). The total population in Finland considering all ages was 5,187,952 in 2000 and 5,390,036 in 2011 [16]. 

The survey participants were interviewed either at a separate home visit, at local health care facilities or by phone, and a health inspection was conducted by a physician during the home visit or at the local health care afterwards. In addition, blood samples for research purposes were collected during the visits and stored frozen at ˗70 °C for later use. Data from patient data registries were obtained to supplement the medical information gathered through the survey.

### Clinical data

The following data were collected with questionnaires and interviews in the present study: age, sex, level of education, employment and current smoking status, doctor visits per year, as well as possible presence of chronic gastrointestinal disease, coronary artery disease, type 1 or 2 diabetes and other autoimmune diseases and malignancy. Information on the presence of celiac disease, other chronic conditions and possible gluten-free diet was either self-reported or based on patient registry data. Autoimmune disease included the following diseases: Type 1 diabetes, psoriasis, rheumatoid arthritis, spondylarthritis, ulcerative colitis, Crohn’s disease, systemic lupus erythematosus, Sjögren’s disease, sarcoidosis and lichen sclerosus; the cause of possible thyroid disease was not specified in the survey, so these conditions were therefore not included under autoimmune diseases. The participants also completed a standard food frequency questionnaire to assess the frequency of food and nutritional consumption. The credibility of the answers was checked by a dietician [14]. The questionnaire was used to identify the presence of special diets including lactose-free, low cholesterol, weight loss, food allergy and vegetarian diets, as well as intake of dietary fibre (g/day). Additionally, body mass index (BMI, kg/m^2^) was recorded.

The assessment of depression, anxiety and general psychological distress was carried out using the validated General Health Questionnaire and the Beck Depression Inventory questionnaires [17, 18]. The psychological status was screened using the General Health Questionnaire, with the total score determining the assessment. The scores are based on a 4-point Likert scale (0-1-2-3), with responses to all items summed up to produce a total score ranging from 0 to 36, with higher scores indicating more psychological distress. Beck Depression Inventory is a 21-item, multiple-choice inventory employing Guttman scaling designed to assess the level of depression in adults. Each item is scored 0 to 3 points, with the total score ranging from 0 to 63, with higher scores indicating more depressive symptoms.

### Laboratory parameters and case definition

Plasma hemoglobin levels were analyzed from the stored samples by THL. Anemia was defined as hemoglobin < 115 g/l in women and < 132 g/l in men according to national reference values for regression analysis [19]. Serum 25-hydroxyvitamin D concentration was analysed using either radioimmunoassay (Health 2000; DiaSorin Inc., MN, USA) or chemiluminescent immunoassay (Health 2011; Abbot Laboratories, IL, USA) [20]. Total cholesterol was measured either enzymatically (Roche Diagnostics, Mannheim, Germany) [12] or by immunoassay (Abbot) [13]. Ferritin was measured in with Chemiluminescent microparticle immunoassay (Abbot) [21]. 

Serum IgA class transglutaminase antibodies and endomysial antibodies were analyzed at Tampere University Celiac Disease Centre by commercial Eu-tTG^®^ (Eurospital S.p.A., Trieste, Italy; Health 2000) or Celikey^®^ (Phadia, Freiburg, Germany; Health 2011). Both assays use human recombinant transglutaminase as an antigen, and the results are given in arbitrary units (AU). Values ≥ 7.0 U/mL for Eu-tTG and ≥ 5.0 U/mL for Celikey were considered positive and further tested for endomysial antibodies. Transglutaminase antibody (Eu-tTG) titers in 2000 between 5.1 and 6.9 U/ml were considered high normal and those equal to and less than 5.0 U/ml low normal. Endomysial antibodies were determined using an indirect immunofluorescence method as described elsewhere [22]. A serum dilution of 1:≥5 was considered positive and positive sample was further diluted up to 1:4000.

PWAG refers to individuals on a gluten-free diet without previously reported diagnosis of celiac disease and negative transglutaminase antibodies and endomysial antibodies. Individuals with either a previously reported diagnosis or positivity for both transglutaminase antibodies and endomysial antibodies were considered to have celiac disease [23], whereas those avoiding gluten with positive transglutaminase antibodies but negative endomysial antibodies had an unclear status and were classified neither as PWAG or celiac disease. The further diagnostic clarification was not possible due to strict Finnish data protection regulations, which limit the access to personal health information.

### Statistics

Descriptive data is given as percentages or as mean and ranges as appropriate. Prevalence figures are given with 95% confidence intervals. For the prevalence and incidence analyses, complex analyses function with provided weights was used in SPSS software (version 27; IBM, New York, United States). The inverse probability weights were used to reduce bias due to non-participation and to provide nationally representative results [15]. Post-stratification weights were calibrated according to age, gender, area and language in 2000. In 2011, the participation rate was lower, and separate weights were created according to participation rate [15]. Statistical comparisons between the study groups were carried out with the chi-square test. In the complex sample analyses, the significance was tested using the adjusted F variant of the second-order Rao-Scott adjusted chi-square statistics. The likelihood for a later PWAG in relation to sex, anemia, transglutaminase antibody levels and autoimmune diseases in subjects without PWAG in 2000 was analyzed with logistic regression to provide odds ratios (OR) with 95% confidence intervals. Analysis of covariance was used to adjust for characteristics. *P* values < 0.05 were considered significant.

## Results

In 2000, 5,777 people, representing 2,682,733 Finnish adults, and in 2011 3,866 people, representing 1,967,876 adults, met the inclusion criteria. Among them, 11 people, representing 4,442, were PWAG in 2000, and 24 people, representing 13,881, were PWAG in 2011. (Fig. 1). In 2000, 33 subjects were found to have diagnosed celiac disease or were seropositive to both transglutaminase antibodies and endomysial antibodies and 78 such subjects in 2011. In addition, two subjects avoiding gluten had positive transglutaminase antibodies in 2011 without celiac disease; none such were found in 2000. celiac disease patients and those seropositive on gluten-free diet were not included in the PWAG group following protocol. The selected characteristics of the PWAG subjects included in the analysis are provided in Table 1.

**Table 1:** Selected weight-adjusted characteristics of the total representative cohort in health 2000 and 2011 surveys included in the present study

	Health 2000, *n* = 2 682 733	Health 2011, *n* = 1 967 876
	%	%
Age, mean (range), years	52 (30–99)	55 (29–97)
Females	53.0	54.3
Higher education	29.4	39.8
Unemployed	7.1	4.2
Coronary artery disease	7.1	5.9
Gastrointestinal disease^1^	12.8	13.2
Celiac disease	0.6	2.0
Type 1 or type 2 diabetes	5.2	7.8
Any malignancy	4.6	7.2
Current smoking	21.5	14.1

^1^ Ulcerative colitis, Crohn’s disease, celiac disease, lactose intolerance, irritable bowel syndrome

The weight-adjusted total prevalence of PWAG increased significantly in Finland from 0.2% in 2000 to 0.7% in 2011 (*p* < 0.001). In separate analysis, a significant increase was observed in subjects aged 30 to 49 and over 70 years, but not in those aged 50 to 69 years (Table 2). No significant difference was noted between men and women (data not shown).

**Table 2:** Weight-adjusted prevalence of people avoiding gluten without Celiac disease in 2000 and 2011 in Finnish population according to age

	Health 2000, *n* = 2 682 733		Health 2011, *n* = 1 967 876		2000 vs. 2011
Age, years	%	95% CI	%	95% CI	*P*
30–49	0.1	0.0–0.3	0.7	0.3–1.3	0.003
50–69	0.2	0.1–0.5	0.5	0.3–1.0	0.060
70-	0.3	0.1–0.9	1.3	0.5–3.3	0.019
All	0.2	0.1–0.3	0.7	0.5–1.1	< 0.001

CI, confidence interval

**Fig. 2:**
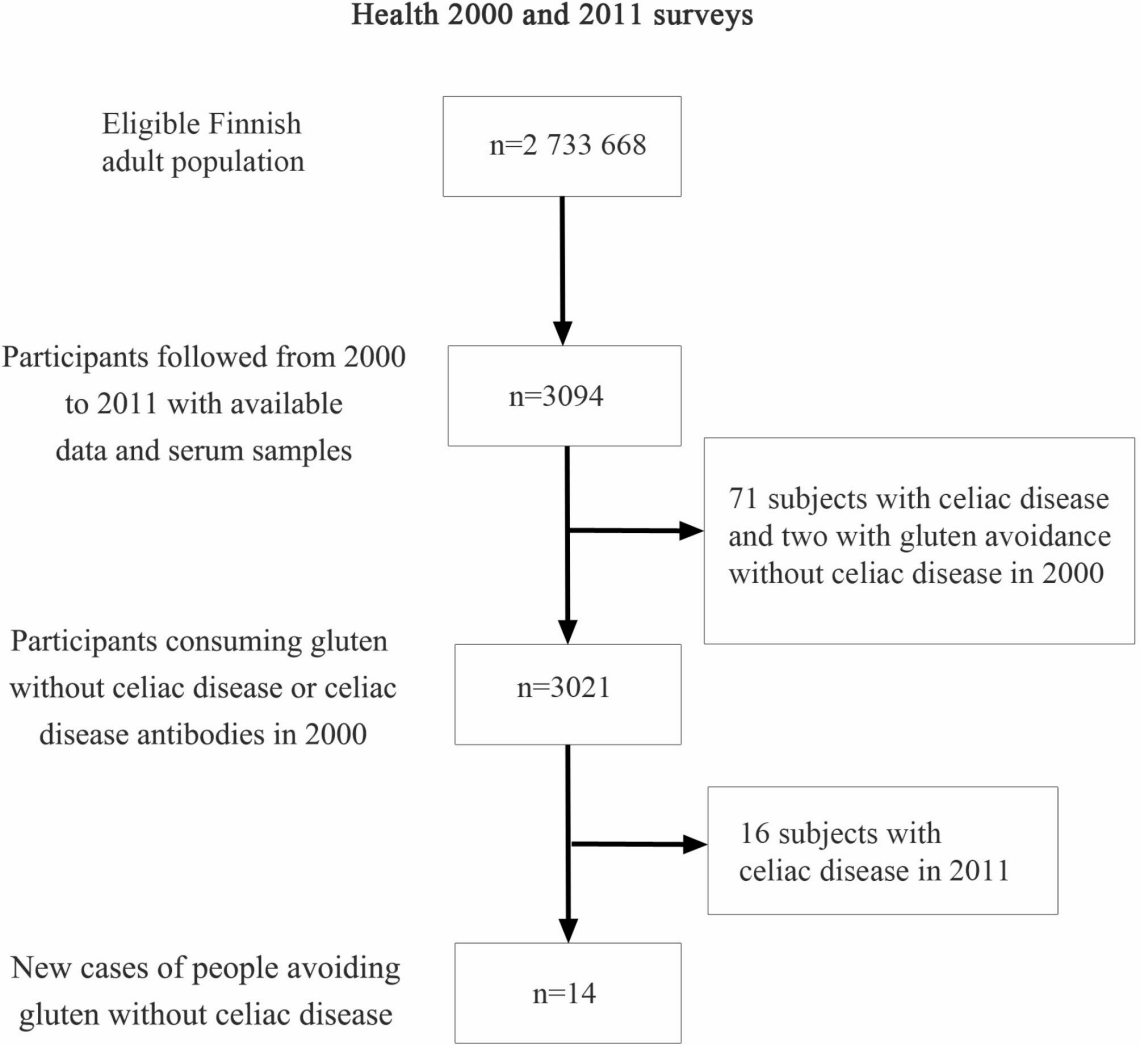
Flowchart of the prospective follow-up cohort. The cohort consisted of individuals who participated in both Health 2000 and Health 2011 surveys, had available data on celiac disease and gluten-free diet and had provided serum sample for the measurement of tissue transglutaminase antibodies. The diagnosis of celiac disease was based on patient´s responses and supplementary medical registry data with addition of those subjects having positivity in both tissue transglutaminase and endomysial antibodies. “People avoiding gluten without celiac disease” refers to subjects who avoid dietary gluten without celiac disease diagnosis and had negative celiac disease antibodies

Celiac disease status, celiac disease serology and gluten diet data were available from 3094 subjects both in 2000 and 2011 and of these 71 had celiac disease and two had PWAG in 2000 in this prospective follow-up cohort. Fourteen of the 3,021 individuals that consumed gluten-containing diet without diagnosed celiac disease or celiac disease antibodies became PWAG between 2000 and 2011 (Fig. 2), resulting in an annual incidence of 42 (95% confidence interval 25–71) per 100.000. Sex (OR 1.43; confidence interval 95% 0.48–3.32) or autoimmune diseases (1.06; 0.14–8.12) did not significantly affect the odds for becoming PWAG. None of the subjects who developed PWAG had anemia, irritable bowel syndrome or ulcerative colitis in 2000. All individuals meeting the criteria of PWAG from 2000 to 2011 had low normal transglutaminase antibody levels below 5 U/l in 2000 as opposed to high normal transglutaminase antibody levels of 5.0 to 6.9. Interestingly, one of the PWAGs in 2000 reported to have received a celiac disease diagnosis between 2000 and 2011.

In addition to gluten-free diet, the PWAG group adhered to lactose-free and food allergy diets more frequently than those not avoiding gluten. In officially diagnosed lactose-intolerance, significant difference was seen only in 2011 (Table 3). Additionally, the PWAG group had less often fulltime employment, more depressive symptoms in Beck Depression Inventory and more irritable bowel syndrome in 2000, but not in 2011, while no significant differences were observed between the PWAG and non-PWAG groups in the other study variables (Table 3). Hemoglobin levels were trending lower in PWAG compared to non-PWAG subjects (*p* = 0.052) in 2000 but ferritin was insignificant (Table 3).

**Table 3:** Comparison of characteristics between people avoiding gluten without Celiac disease (PWAG) and non-PWAG individuals in 2000 and 2011

	Health 2000					Health 2011				
	PWAG *n* = 11		Non-PWAG *n* = 5733			PWAG *n* = 24		Non-PWAG *n* = 3764		
	%		%		*P*	%		%		*P*
Females	81.8		55.4		0.102	66.7		56.0		0.294
Fulltime employment	18.2		52.6		0.022	41.7		48.5		0.504
Lactose-free diet	36.4		9.2		0.004	41.7		12.0		< 0.001
Low cholesterol diet	9.1		7.7		0.852	8.3		6.6		0.731
Weight loss diet	0		2.8		0.533	4.2		1.6		0.327
Food allergy diet	9.1		1.9		0.100	12.5		3.0		0.007
Vegetarian diet	0		0.3		0.861	4.2		1.2		0.185
IBS	9.1		1.9		0.026	4.2		1.8		0.420
IBD	0		2.3		0.613	0		1.8		0.513
Asthma	18.2		8.8		0.342	16.7		9.8		0.225
Autoimmune disease^1^	0		8.2		0.345	4.2		6.4		0.655
Lactose-intolerance	9.1		7.6		0.853	29.2		7.6		< 0.001
Depression	18.2		9.2		0.303	4.2		8.0		0.513
	Mean	SD	Mean	SD	*P*	Mean	SD	Mean	SD	*P*
Age, years	60.8	20.6	52.6	14.9	0.087	56.0	16.3	56.1	13.5	0.978
BMI, kg/m^2^	28.3	8.4	26.9	4.6	0.455	25.8	4.1	27.1	4.7	0.195
School, years	9.1	4.7	11.3	4.1	0.321	12.1	3.8	13.0	4.1	0.243
Dietary fiber, g/day	26.2	13.2	25.1	10.5	0.755	23.5	12.4	25.6	11.0	0.357
Doctor visits, n/year^2^	3.9	2.3	4.1	4.1	0.761	5.7	4.0	3.7	3.6	0.027
Hemoglobin, g/l	131	20.0	143	14.4	0.052	NA	NA	NA	NA	NA
Ferritin, µg/l	64.2	75.5	97.8	113.1	0.652	NA	NA	NA	NA	NA
TGA, U/l or %^3^	2.2	1.4	2.4	2.3	0.834	0^3^	NA^2^	5.0^3^	NA^3^	NA^3^
Cholesterol, mmol/l	6.1	1.1	5.9	1.1	0.859	5.7	1.1	5.4	1.0	0.165
D-25-vitamin, nmol/l	43.1	16.8	45.3	16.9	0.659	82.8	18.8	76.2	22.3	0.152
BDI, points	12.3	9.8	7.1	6.9	0.035	2.3	3.5	2.7	3.8	0.604
GHQ-12, points	3.5	4.2	1.8	2.9	0.085	1.8	2.9	1.5	2.6	0.663

All data is sex- and age adjusted when applicable. ^1^ Type 1 diabetes, psoriasis, rheumatoid arthritis, spondylarthritis, ulcerative colitis, Crohn’s disease, systemic lupus erythematosus, Sjögren’s disease, sarcoidosis and lichen sclerosus. Data were available from > 90% subjects, ^2^except in non-PWAG doctors’ visits with 4244 in 2000 and 1154 in 2011. ^3^ In 2011, transglutaminase antibody values were reported only qualitatively and thus, comparison of serological titers below normal was not possible. None of the subjects in the PWAG group and 19 (5.0%) in the non-PWAG group exhibited transglutaminase values above normal. BDI, Beck´s depression inventory; BMI, body mass index; GHQ, general health questionnaire-12; IBD, inflammatory bowel disease; IBS, Irritable bowel syndrome; NA, not available; TGA, tissue transglutaminase antibodies

## Discussion

The prevalence of PWAG in Finland was 0.2% in 2000 and increased to 0.7% in 2011, demonstrating an almost quadrupling of the cases within a decade. The annual incidence rate was 49 per 100,000 persons. For comparison, we previously observed the prevalence of celiac disease to increase from 2.1 to 2.4% during the same time [23]. Overall, based on these studies, more than 3% of the Finnish population is following a gluten-free diet. There was no linear change in the prevalence with age, and and no significant difference was observed between sexes. However, in line with Choung et., women were trending towards significance in the PWAG groups both in 2000 and 2011 [8]. 

No comparable studies involving nationwide representative cohorts with such long follow-up period are available; however, some previous studies have also investigated variations in the prevalence of PWAG over time. Choung et al. observed an increase from 0.5% in 2009–2010 to 1.7% in 2013–2014 in the National Health and Nutrition Examination Survey (NHANES) cohort in USA [8]. Other studies have reported even higher figures. For example, a Dutch study reported a prevalence of 6.2% in 2015. It was based on a questionnaire distributed to adults visiting dental practices, markets and university facilities [6]. In a corresponding UK study, the prevalence of gluten sensitivity reached 13% among individuals recruited outside shopping malls and transportation stations in 2012 [5]. An Australian study found that the prevalence of wheat avoidance was 7.3% in a cross-sectional survey mailed to randomly selected adults in 2010-11 and another Australian study found a prevalence of about 14% in 2015 and 2018 in a general population cohort [7, 24]. Similar high variation in the reported figures in genetically alike populations have not been observed in celiac disease, and there is no obvious biological explanation. These inconsistencies may reflect methodological and sampling variations, cultural differences, as well as the subjectivity of the PWAG diagnosis. Overall, comparison between studies is hampered by limited geographical coverage, ethnically and demographically diverse study cohorts, and the use of different criteria [5, 6, 24]. 

We found a relatively high incidence of PWAG of 42 per 100.000 without clear causing factors as to subject characteristics or prior diseases, however, the probable cause could be the general increased awareness of possible gluten related symptoms through media etc [3]. Interestingly, these figures are quite similar to the celiac disease figures of 45 per 100.000 persons in Finland [23]. Much higher incidence was observed in a questionnaire study by Potter et al., where 5.3% of subjects developed gluten sensitivity between 2015 and 2018 with an annual rate of 1.8% equalling numbers up to 1800 per 100,000 persons [24]. The incidence numbers seem thus to be currently rather high, however, it remains to be seen whether this is a continuing or a momentary phenomenon.

The presence of anemia, pre-existing autoimmune diseases, or possibility of underlying celiac disease, in the form of high normal transglutaminase antibody levels, were not associated with the likelihood of developing PWAG, despite being linked to the condition in some previous studies [8–11]. Nevertheless, it must be noted that one individual in the PWAG group in 2000 had received a celiac disease diagnosis later. Unfortunately, due to the strict data protection regulations, we were unable to determine how the diagnosis was established. Notably, all new PWAG cases that emerged between 2000 and 2011 exhibited transglutaminase antibody levels within the low normal range in 2000. This was important because we have previously observed that high normal transglutaminase antibody values may significantly increase the risk of developing celiac disease in the future [8]. The lack of serological and autoimmune connections, along with the independent epidemiological burden and variation in prevalence [25], provides further evidence that PWAG is a condition distinct from the HLA-dependent and immune-mediated celiac disease. In fact, one potential bias in the studies investing PWAG is the presence of unrecognized celiac disease, which may partly explain the previously observed associations with serology and autoimmunity.

Although the aforesaid diagnostic challenges and methodological variations make it difficult to estimate the exact prevalence of PWAG, findings within the same study cohorts still demonstrate that the number of cases has increased rapidly [5, 6, 24, 25]. Although true biological causes cannot be fully excluded, it is possible that the increase is at least partially explained by the hype surrounding the perceived health benefits of dietary gluten avoidance. This may be further exacerbated by the active marketing and advertising of these products [2, 26, 27]. There is likely a significant overlap with irritable bowel syndrome, which may lead to unwarranted assumptions about the benefits of a gluten-free diet, particularly if the diet simultaneously results in low consumption of fermentable oligosaccharides, disaccharides, monosaccharides, and polyols [28, 29]. Supporting this, there was a significantly more PWAG subjects with diagnosed irritable bowel syndrome in 2000, with 9.1% of PWAGs having the diagnosis compared to 1.9% in non-PWAGs (*p* = 0.026), though the link was non-significant in 2011. Furthermore, double-blind challenge studies have indicated that gluten does not appear to be the primary cause of symptoms in PWAG; rather, other components, including oligosaccharides, may play a more significant role [28, 30]. Furthermore, given that the effects of gluten or other potentially harmful nutritional factors may be dose-dependent, with some individuals exhibiting greater tolerance than others, a personalized nutritional plan might be needed to optimize treatment and avoid nutritional deficiencies [1, 28–30]. 

Emphasizing the role of patient-related factors, the PWAG group exhibited more depressive symptoms based on the Beck Depression Inventory, less full-time employment in 2000, and visited the doctor more often in 2011 compared to those not avoiding gluten [5, 6, 31]. Anxiety and depression have been linked to the avoidance of gluten-containing without celiac disease foods also in previous studies [31] and may in part contribute to the observed lower employment rates among PWAG. Additionally, in line with previous studies [5, 6], they adhered more often to additional food restrictions beyond a gluten-free diet. This can be problematic, as multiple dietary restrictions increase the risk of nutrient deficiencies [25] and may further exacerbate health challenges in a vicious cycle. Another important patient group are children avoiding gluten without celiac disease, for whom nutritional issues related to such diets could even be developmentally harmful, and the potential benefits and risks in such cases should be carefully assessed [26, 27, 32]. The Health 2000 and 2011 surveys included only adults, highlighting the need for further research in pediatric cohorts. Overall, regardless of age, the PWAG group could benefit from intensified healthcare support and dietary counselling.

The main strengths of the present study include the availability of large, nationally representative cohorts, various individual-level data, and the use serological tests to exclude celiac disease. Limitations included a lower participation rate in 2011 compared to 2000, which, according to feedback, was caused by a lack of suitable time and place for appointments, as well as insufficient information gained about their own health during the study [13]. Several efforts were made to increase the participation rate, and adjustments were implemented to ensure a representative population analysis [13]. In addition, the data lacked information on HLA genotype and family risk of celiac disease. Furthermore, due to the strict data protection regulations, the data regarding individual study participants was not available afterwards for confirmatory analyses. An additional limitation was the somewhat outdated nature of the data and the modest number of subjects with PWAG. Future studies on PWAG should thus aim to include more up-to-date, nationally representative data.

To conclude, the prevalence of PWAGs almost quadrupled between 2000 and 2011 in a nationwide population-representative adult cohort. The rapid increase in prevalence and lack of association with common celiac disease related risk factors suggests that these individuals have a condition distinct from celiac disease. The increased psychosocial burden and the presence of multiple dietary restrictions emphasize the need for intensified healthcare support and dietary counselling. More research is needed to decipher the pathological mechanisms and the true causal role of gluten in PWAG [2, 4, 26, 28, 30, 32]. 

## Data Availability

The data that support the findings of this study are available from Finnish Institute for Health and Welfare. Restrictions apply to the availability of these data, which were used under license for this study. Data are available at: https://thl.fi/en/research-and-development/thl-biobank; with the permission of Finnish Institute for Health and Welfare.

## Abbreviations

PWAG: People avoiding gluten without celiac disease
